# Antitumour activity of a potent MEK inhibitor RDEA119/BAY 869766 combined with rapamycin in human orthotopic primary pancreatic cancer xenografts

**DOI:** 10.1186/1471-2407-10-515

**Published:** 2010-09-28

**Authors:** Qing Chang, Mark S Chapman, Jeffrey N Miner, David W Hedley

**Affiliations:** 1 Division of Applied Molecular Oncology, Ontario Cancer Institute/Princess Margaret Hospital, University of Toronto, Toronto, Ontario, Canada; 2 Research and Development, Ardea Biosciences, Inc., 4939 Directors Place, San Diego, CA, USA; 3 Department of Medical Oncology and Hematology, Princess Margaret Hospital, Toronto, Ontario, Canada

## Abstract

**Background:**

Combining MEK inhibitors with other signalling pathway inhibitors or conventional cytotoxic drugs represents a promising new strategy against cancer. RDEA119/BAY 869766 is a highly potent and selective MEK1/2 inhibitor undergoing phase I human clinical trials. The effects of RDEA119/BAY 869766 as a single agent and in combination with rapamycin were studied in 3 early passage primary pancreatic cancer xenografts, OCIP19, 21, and 23, grown orthotopically.

**Methods:**

Anti-cancer effects were determined in separate groups following chronic drug exposure. Effects on cell cycle and downstream signalling were examined by flow cytometry and western blot, respectively. Plasma RDEA119 concentrations were measured to monitor the drug accumulation *in vivo*.

**Results:**

RDEA119/BAY 869766 alone or in combination with rapamycin showed significant growth inhibition in all the 3 models, with a significant decrease in the percentage of cells in S-phase, accompanied by a large decrease in bromodeoxyuridine labelling and cell cycle arrest predominantly in G1. The S6 ribosomal protein was inhibited to a greater extent with combination treatment in all the three models. Blood plasma pharmacokinetic analyses indicated that RDEA119 levels achieved *in vivo *are similar to those that produce target inhibition and cell cycle arrest *in vitro*.

**Conclusions:**

Agents targeting the ERK and mTOR pathway have anticancer activity in primary xenografts, and these results support testing this combination in pancreatic cancer patients.

## Background

The Ras-Raf-MEK-ERK signalling network has been the subject of intense research and pharmaceutical scrutiny to identify novel target-based approaches for cancer treatment due to its key role in cancer progression [1]. Activating mutations of K-ras are the earliest consistently detected abnormality in the development of pancreatic cancer, and pancreatic cancers that spontaneously develop in mice with genetically-modified K-ras show similar features to those seen in patients [2]. Aberrant expression of receptor tyrosine kinases such as EGFR and c-Met, and loss of the ERK phosphatase DUSP6 occur during cancer progression and activate the ERK pathway [3]. The ERK pathway can activate genes involved in cell growth and survival, and also regulate metabolic processes including protein translation. An abundant literature has shown that MEK inhibition can enhance the effects of other signalling pathway inhibitors or conventional cytotoxic drugs [1,4,5].

RDEA119/BAY 869766 is a selective, orally available MEK inhibitor. It was selected for clinical development because of its potency and favourable pharmacokinetic profile. RDEA119 is currently undergoing phase I clinical trials in late-stage cancer patients refractory or intolerant to other anticancer therapies [6]. We recently reported on the effects of combined MEK and mTOR inhibition *in vitro *or in xenograft models established from pancreatic cancer cell lines [7]. However, treatments that are effective against pancreatic cancer cell line models often show much less activity in the clinic. Primary xenografts established from patient pancreatectomy specimens and grown at the orthotopic site show typical histological features of pancreatic cancer [8-10], and therefore offer the opportunity for the near-clinical testing of novel molecular targeted agents in a controlled laboratory setting that allows detailed analysis of the relationships between the tumour characteristics, pharmacological effects, and anticancer effects.

In the current study, we tested the effects of RDEA119 as a single agent, or combined with rapamycin in a panel of early-passage primary pancreatic cancer xenografts, grown orthotopically. Acute dosing strongly inhibited tumour proliferation, and chronic treatment produced significant growth inhibition consistent with effects on downstream signalling pathways.

## Methods

### Establishment of primary pancreatic cancer xenografts

Animal experiments were carried out using protocols approved by University Health Network Animal Welfare Committee. The establishment of the primary pancreatic cancer xenografts was done as previously described [8,9,11]. Fresh pancreatectomy samples that were superfluous to diagnostic needs were obtained from the University Health Network Tumour Tissue Bank according to institutional human ethical guidelines. Primary xenografts were established at the orthotopic site of 4-to 5-week-old mice by attaching tumour fragments to the surface of the exposed pancreas by a small incision in the upper left abdomen under general anaesthesia. Three orthotopic primary pancreatic cancer xenografts, designated as OCIP 19, 21, and 23, were used for these experiments.

### Drug preparation and treatment protocols

The MEK inhibitor RDEA119/BAY869766 was provided by Ardea Biosciences, Inc. (San Diego, CA). Rapamycin was purchased from Calbiochem (San Diego, CA). Rapamycin was dissolved in DMSO at 1 mg/ml, aliquoted, and stored at -20°C. RDEA119, which has good oral bioavailability, was prepared freshly at 3.125 mg/ml in 10% Cremophor EL in saline, for oral gavage *in vivo*.

The 48 h combination therapy experiment included a total of 12 OCIP23 tumour-bearing mice with 3 animals randomly assigned to one of four groups: drug-vehicle control (10% Cremophor EL in saline, oral gavage; DMSO, i.p.), RDEA119 (6.25 mg/kg, oral gavage, b.i.d.), rapamycin (2 mg/kg, i.p.), and RDEA119 plus rapamycin groups (6.25 mg/kg, oral gavage, b.i.d. and 2 mg/kg, i.p., respectively). All mice were sacrificed 48 h after beginning the experiment (4 h after the final dose of RDEA119).

The chronic dosing combination therapy experiment included 36 tumour-bearing mice for each model, with 9 animals randomly assigned to one of four groups: drug-vehicle control, RDEA119, rapamycin, and RDEA119 plus rapamycin groups. In this experiment RDEA119 was administered 6.25 mg/kg, oral gavage, b.i.d. 5-day on and 2-day off, rapamycin was administered 2 mg/kg i.p. once weekly. Due to the different growth rates of these 3 models, the drug administration was initiated on Day 52, 24, and 12 after implantation in OCIP19, OCIP21, and OCIP23, respectively. Animal body weight, and tumour condition were recorded thrice weekly for the duration of the study. After 2-4 weeks of treatment, the animals were sacrificed and the tumours were harvested immediately (4 h after the final dose of RDEA119 or/and 48 h after the final dose of rapamycin). Depending on the subsequent analyses, all harvested tumours were cut into pieces, either snap frozen in liquid nitrogen, homogenized in lysis buffer as for the cell lysates, fixed in 10% formalin for 24 h and paraffin embedded.

### Immunoblotting and quantification

Minced tumour pieces were homogenized in 1 ml lysis buffer. Equivalent amounts of protein were separated on 12% SDS-PAGE gels. Proteins were transferred to polyvinylidene difluoride membranes (Millipore, Bedford, MA) and probed with the appropriate antibodies. Rabbit monoclonal antibodies against phospho-4E-BP1 (Thr37/46) (236B4), rabbit polyclonal antibodies against phospho-p44/42 MAPK (Thr202/Tyr204), phospho-S6 Ribosomal Protein (Ser235/236), phospho-S6 Ribosomal Protein (Ser240/244) and antibodies directed against their nonphosphorylated counterparts, were purchased from Cell Signalling Technology, Inc. (Danvers, MA). The secondary antibodies for western blot (anti-mouse and anti-rabbit IgG antibodies) were from Amersham Biosciences (Buckinghamshire, United Kingdom). Membranes were developed with enhanced chemiluminescence (ECL) Plus detection reagents and imaged using a Typhoon™9410 system (GE Healthcare, Piscataway, NJ). Quantification of immunoblots was performed using ImageQuant 5.2 software by calculating an index based on signal intensity multiplied by signal area.

### Flow cytometric analysis

To study the effect of RDEA119 or/and rapamycin on the cell cycle, snap frozen tumours were minced and permeabilized with 0.1% Triton X-100 and stained with 50 μg/ml propidium iodide (PI)/RNase A (1 mg/ml). DNA histograms were analyzed using ModFit LT™(Verity, Topsham, ME). To determine the effect of RDEA119 or/and rapamycin administration on DNA synthesis in tumour cells *in vivo *and on tumour kinetics, tumour-bearing mice were injected i.p. with 100 mg/kg 5-bromo-2'-deoxyuridine (BrdU) (Sigma Chemical Co., St. Louis, MO) dissolved in PBS 30 min before the mice were sacrificed and the tumour removed. Single-cell suspensions from tumours were prepared by an enzymatic technique for dual label flow cytometry as described previously [12]. Briefly, single-cell suspensions were fixed in 80% ethanol, denatured, neutralized and then stained with anti-BrdU (PRBQD-Alexa 488, Phoenix Flow Systems, Inc., San Diego, CA) and 1 μg/ml 4', 6-diamidino-2-phenylindole (DAPI). BrdU labelling index was analyzed using WinList™6.0 (Verity, Topsham, ME).

### Immunofluorescent, immunohistochemical staining and image analysis

Serial sections were cut from paraffin-embedded tumour tissue: one of these was stained with H&E for transmitted light microscopy and used for selecting the tumoural areas, on which the further image analysis was to be done. The 48h-treatment sections were labelled with primary antibodies against phosphorylated ERK (Cell Signalling Technology, Inc., Danvers, MA) and BrdU (Clone IU-4, MD5000; Caltag Laboratories, Inc., Burlingame, CA). Secondary antibodies used alone were control for nonspecific background. All these sections were counterstained with 1 μg/ml DAPI to outline the nuclear area. p53 (DO-7, Vector Laboratories, Inc., Burlingame, CA) and phosphorylated ERK (Cell Signalling Technology, Inc., Danvers, MA) were stained for transmitted light microscopy for all the chronic dosing sections.

The analysis included the entire viable tumoural area identified on the slide, using a scanning autostage to create composite images of individual fields at 20 × magnification (2.24 mm^2^) as described previously [13]. A tumoural map that included only viable tumour was made using the H&E sections and then carefully adjusted on the composite DAPI images. For each marker, we measured at least two variables: the intensity of the signal (integrated absorbance) and the percentage of positive stained area/nuclei, based on the binary images.

### Analysis of plasma RDEA119 concentrations

Plasma samples were harvested 4 h after the final dose of RDEA119 or/and 48 h after the final dose of rapamycin. Plasma samples were analyzed for RDEA119 using a liquid chromatography-tandem mass spectrometry (LC-MS/MS) method [6]. The method involved the addition of the internal standard ([13C6]RDEA119), protein precipitation with acetonitrile, and final analysis by high-performance LC-MS/MS. An API 5000 triple quadruple mass spectrometer was used to monitor the precursor!product ion transitions of m/z 573!394 and m/z 579!400 for RDEA119 and [13C6]RDEA119 in positive electrospray ion mode. The calibration curves covered the concentration range from 10 to 10,000 ng/ml.

### Statistical analysis

Data from quantitative experiments are presented as mean ± SE. Data obtained from the xenograft models (tumour weight and animal body weight) and comparisons between four treatment groups were analyzed using a repeated measure one-way ANOVA test with Newman-Keuls multiple comparison post test. *P *< 0.05 was considered statistically significant.

## Results

### Characterization of orthotopic primary pancreatic cancer xenografts

**Figure 1:**
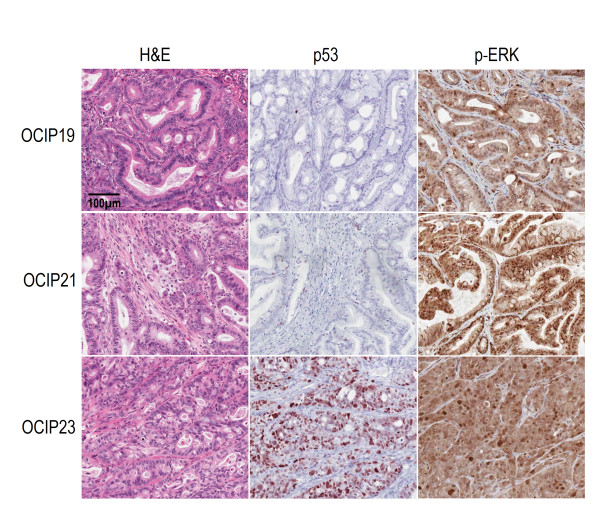
**Characterization of orthotopic primary pancreatic cancer xenografts**. Representative H&E and immunohistochemical staining of p53 and phosphorylated ERK in 3 orthotopic primary pancreatic cancer xenografts. The scale bar represents 100 μm.

Histological examination of the H&E sections showed that the primary xenografts were adenocarcinomas with features similar to the original surgical specimens. The histological features were more complex than those typically seen in xenografts established at the subcutaneous site from cell lines, as has been previously noted [14]. As reported in our previous studies [11], the OCIP19 and OCIP21 tumours are relatively slow growing, and by histology moderately well differentiated with mucin production. OCIP21 has a wild type K-ras, whereas OCIP19 is ~60% K-ras mutant (Codon 12). Both of these models have wild type p53. In contrast, OCIP23 is 100% K-ras mutant (Codon 12), p53 mutant, poorly differentiated, and more rapidly growing. Phosphorylated ERK was readily detected in all of the 3 models (Figure 1).

### Effects of RDEA119 plus rapamycin combination on tumour growth in orthotopic primary pancreatic cancer xenografts

**Figure 2:**
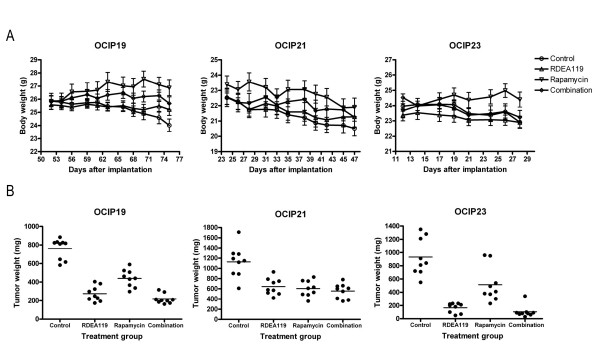
**Effects of RDEA119 plus rapamycin combination on tumour growth**. Effects of RDEA119 plus rapamycin combination on tumour growth in 3 orthotopic primary pancreatic cancer xenografts. A. Body weight during the chronic administration of vehicle control, single agents, and concurrent combination. Due to the different growth rates of these 3 models, the drug administration was initiated on Day 52, 24, and 12 after implantation in OCIP19, 21, and 23, respectively. RDEA119 was administrated at 6.25 mg/kg p.o. b.i.d. (5-day on, 2-day off) and rapamycin at 2 mg/kg i.p. once weekly for 2-4 weeks; all the treatments were well tolerated. Error bars are SEM. B. Tumour weights at the completion of chronic dose administration. Horizontal lines indicate the mean value for each group.

Twice daily oral administration of RDEA119 at a dose of 6.25 mg/kg was well tolerated in the tumour-bearing mice. Loss of weight occurred in the control groups of all the three models, which was presumably the effect of the tumours. In the OCIP19 tumour-bearing mice, the rapamycin treatment group significantly gained weight (*P *< 0.001 vs. Control; *P *< 0.001 vs. Combination; *P *< 0.001 vs. RDEA119). There were no statistically significant differences in the body weights of RDEA119, or combined with rapamycin treated and control animals at the end of treatment in any of the 3 models (Figure 2A). Because of the location of the tumour in the orthotopic pancreas site, the tumour size was not measured until the end of the experiments when the animals were killed 4 h after the final dose. Consequently, at the end of the 2-to 3-week treatment, orthotopic tumours were dissected free of surrounding normal tissues and weighed. As shown in Figure 2B, RDEA119, rapamycin or combination treatment of tumour models OCIP19, 21, and 23 resulted in statistically significant delay in tumour growth when compared with vehicle-treated controls (*P *< 0.001). There was significant difference between rapamycin with RDEA119 single agent treatment group (*P *< 0.001) or combination group (*P *< 0.001) in OCIP19 and OCIP23, whereas there was no significant growth inhibition difference between RDEA119 single agent treatment group with the combination group in all the 3 models.

### Cell cycle effects *in vivo*

**Figure 3:**
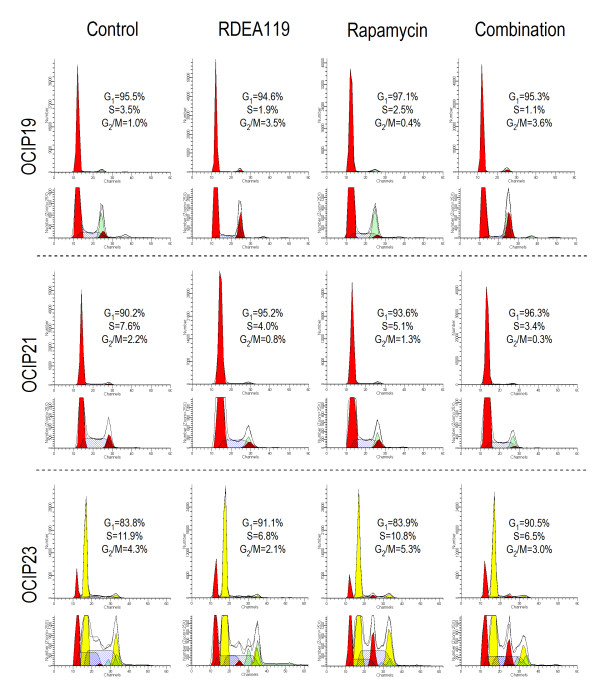
**Cell cycle effects *in vivo***. Representative single parameter DNA histograms showed cell cycle effects following chronic dose administration of 3 orthotopic primary pancreatic cancer xenografts. Percent cells in G_1_, S and G_2_/M phases are given. For OCIP23, which is aneuploid, the values are for the cancer cells.

Cellular DNA content analysis by flow cytometry was used to determine the cell cycle effects following RDEA119, rapamycin or combination administration (Figure 3). OCIP19 and OCIP21 are near-diploid, and the mouse and human G_1 _peaks overlap, whereas OCIP23, which is the most aggressive growing model, is aneuploid. The percentage of S-phase in each model was significantly decreased following the treatment of RDEA119 or the combination with rapamycin in all the 3 models (*P *< 0.001).

### Effects of 48 h combined inhibition of MEK and mTOR on tumour proliferation

**Figure 4:**
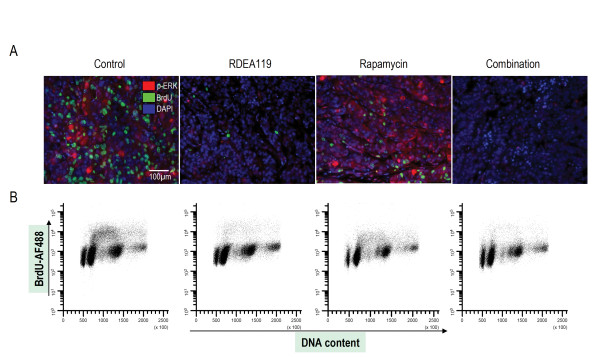
**Effects of 48 h combined inhibition of MEK and mTOR on tumour proliferation**. Effects of 48 h combined inhibition of MEK and mTOR on tumour proliferation. A. Dual fluorescence image analysis of BrdU and phosphorylated ERK in OCIP23 xenograft models. Representative images (20 × single fields) of tissue sections from OCIP23 xenografts following 48 h treatment stained for phosphorylated ERK (red), BrdU (green) and counterstained for DAPI (blue). The scale bar represents 100 μm. B. BrdU incorporation was measured by flow cytometry in OCIP23 tissue post a 48 h exposure to 6.25 mg/kg b.i.d. RDEA119 and 2 mg/kg rapamycin.

The OCIP23 xenograft model was selected for further study of the 48 h treatment effects due to its genetic features and aggressive growth. As shown in Figure 4A, dual immunofluorescent images of phosphorylated ERK and BrdU revealed that 48 h treatment of RDEA119 or combination produced a large decrease of phosphorylated ERK and BrdU labelling. As shown in Figure 4B, there was a large decrease in BrdU uptake following the 48 h treatment, indicating that RDEA119 results in the cell cycle arrest predominantly in G_1_.

### Effects of RDEA119 plus rapamycin combination on downstream signalling

**Figure 5:**
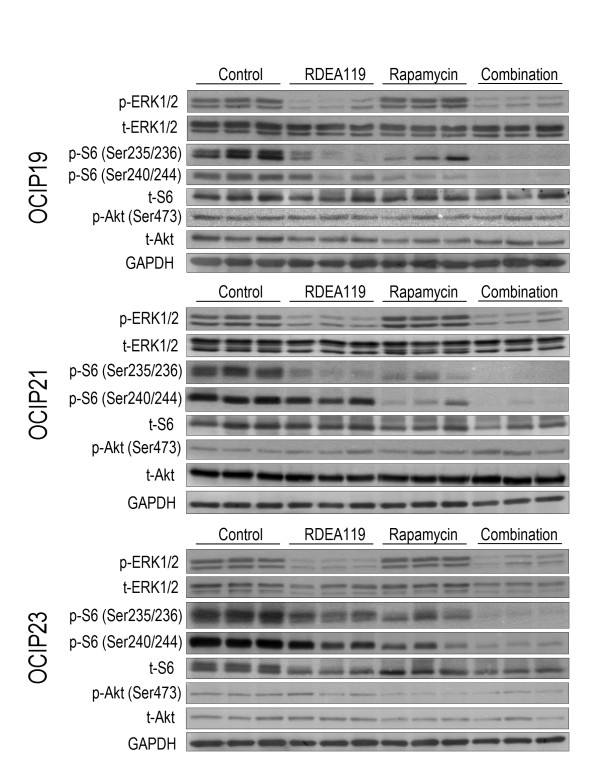
**Effects of RDEA119 plus rapamycin combination on downstream signalling**. Tumour lysates obtained 4 h after the final dose of RDEA119 or/and 48 h after the final dose of rapamycin in the chronic dosing groups of animals were analyzed by western blot using the indicated antibodies. Representative blots showed the effect of combined inhibition of MEK and mTOR on downstream signalling.

**Figure 6:**
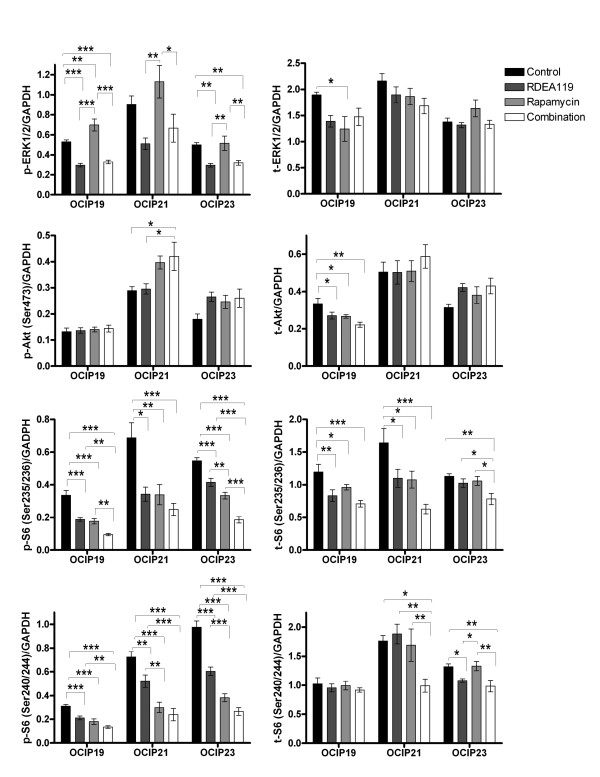
**Immunoblotting quantification**. Western blot signals were quantified and intensity of phosphorylated and total protein normalized loading control GAPDH was presented (n = 9 for each group). Error bars are SEM. *P < 0.05; **P < 0.01; ***P < 0.001.

Representative western blots are shown in Figure 5, and quantitative graphs in Figure 6. RDEA119, either alone or in combination, notably dephosphorylated ERK1/2 in OCIP19 and OCIP23 (*P *< 0.001, RDEA119 vs. Control, Combination vs. Control in OCIP19; *P *< 0.01, RDEA119 vs. Control, Combination vs. Control in OCIP23), a similar trend was also seen in OCIP21. In OCIP19, rapamycin significantly increased ERK1/2 phosphorylation (*P *< 0.01 vs. Control), and a similar trend was also seen in the other 2 models. The levels of Ser473 phosphorylated Akt were significantly increased following the combination drug administration in OCIP21 (*P *< 0.05). Treatment with rapamycin produced decreased phosphorylation of both pairs of serine sites on S6 ribosomal protein (Ser235/236 and Ser240/244), consistent with previous studies [7,15]. The combination treatment of RDEA119 and rapamycin produced the greatest decrease in Ser235/236 and Ser240/244 phosphorylated S6 ribosomal protein in all the 3 models. It should also be noted that significant decreases of total proteins occurred in some cases following the chronic dosing treatment.

### Plasma RDEA119 concentrations in 3 xenograft models

Compound concentrations in the plasma of RDEA119 single agent or combination groups 4 h after last treatment were in average 3052 vs. 3137 ng/ml, 3094 vs. 3067 ng/ml in OCIP19 and OCIP21, respectively. Lower plasma RDEA119 concentrations in the combination group (in average 1657 ng/ml) compared to RDEA119 single agent treatment group (in average 2833 ng/ml) were observed in OCIP23.

## Discussion

It is likely that the use of orthotopically grown early passage primary xenografts provides a better prediction of clinical activity compared to subcutaneous xenografts derived from cell lines. OCIP19, 21, and 23 were selected for this study to represent the spectrum of pancreatic cancer, based on their genetic features and growth rates.

The anti-cancer effects of RDEA119 alone or in combination with rapamycin were tested using twice daily oral dosing combined with once weekly i.p. The RDEA119 dosing is similar to the treatment schedules commonly used in clinical trials testing novel molecular targeted agents. The primary end points for this part of study were animal body and tumour weight at the end of treatment period. The tumour-bearing mice were also monitored by abdominal palpation during the treatment, which gave a rough indication of tumour growth. No significant spontaneous metastases were observed at the end of the treatment. Using 6.25 mg/kg of RDEA119 twice daily for 5 days per week dose schedule or combined with 2 mg/kg rapamycin once weekly dose schedule, no increased toxicity relative to the drug vehicle control group during the treatment period. In all the three primary xenograft models, we observed a statistically significant reduction in the tumour weight relative to the drug vehicle control group, which was consistent with the impression obtained from the abdominal palpation that oral twice daily treatment with RDEA119 or combined with rapamycin inhibited tumour growth. Rapamycin also gave statistically significant growth inhibition in all three models, and this effect appeared to be additive, rather than synergistic, when the two agents were combined. OCIP23, which is the most aggressive growing model, showed the best response to the RDEA119 single agent or the combination treatment.

The cell cycle effects revealed by DNA content analysis and BrdU labelling were consistent with the tumour growth inhibition effects and suggest that the ERK pathway plays a major role driving proliferation in pancreatic cancer. Next, the expression and constitutive phosphorylation of molecules involved in the ERK and mTOR downstream signalling pathways in pancreatic cancer tissues were examined. Considerable inter-tumoural heterogeneity within the replicate samples was observed, consistent with our previous studies [11]. There are several mechanisms involved in the negative regulation of Akt activity by mTORC1, the effect of S6K1 on IRS-1 downstream of IGF-1 and/or insulin receptors, or other growth factors [16,17]. For example, in the present study we observed activation of ERK following rapamycin treatment in OCIP19 and 21, and the trend of activation of Akt with dual MEK/mTOR inhibition in all the 3 models, which is consistent with feedback regulation of ERK and Akt [4,18]. S6 ribosomal protein phsophorylation at Ser236/236 and Ser240/244 was notably greater inhibited with combination treatment all the 3 models. S6 is more likely responsive to rapamycin rather than RDEA119 in all the 3 models, which is consistent with our previous findings [7] that drug sensitivity might be affected by the tumour microenvironment *in vivo*.

Induction of Bim expression with RDEA119 or combined with rapamycin was also observed by western blot in all the 3 models (data not shown), indicating that Bim could account, at least in part, for the possible mechanisms that causes cell death. It is consistent with the previous reports on the other MEK inhibitors [19,20]. In spite of the complexity of inter-tumoural heterogeneity in the prediction of *in vivo *response, the combination of agents targeting the ERK and mTOR pathway has anticancer activity in primary pancreatic cancer xenografts. This effect was seen in the K-ras and p53 mutant OCIP23 model, as well as the less aggressive models.

## Conclusions

The current tendency is to combine molecular targeted agents with the nucleoside analogue gemcitabine in clinical trials treating patients with advanced pancreatic cancer. However, the inhibition of BrdU uptake into DNA during exposure to RDEA119 or its combination with rapamycin cautions that concurrent administration with gemcitabine might be antagonistic, and further investigation of treatment schedules combining these agents appears warranted. Alternatively, given the apparent low toxicity of the RDEA119 + rapamycin combination, it might have palliative benefit for patients with chemotherapy-refractory pancreatic cancer.

## List of abbreviations

ECL: enhanced chemiluminescence; PI: propidium iodide; BrdU: 5-bromo-2'-deoxyuridine; DAPI: 4', 6-diamidino-2-phenylindole.

## Competing interests

MSC and JNM are employees of Ardea Biosciences.

## Authors' contributions

QC designed the study, carried out the experiments described in the study and drafted the manuscript. MSC, JNM, and DWH conceived, reviewed the study and revised the manuscript. All authors read and approved the final manuscript.

## Pre-publication history

The pre-publication history for this paper can be accessed here:

http://www.biomedcentral.com/1471-2407/10/515/prepub
